# Intraoperative vascular air embolism and intracardiac thrombosis complicating liver transplantation: a case report

**DOI:** 10.1186/s13256-024-04376-8

**Published:** 2024-02-18

**Authors:** Jorge Sinclair De Frías, Lorenzo Olivero, Zachary Fleissner, Justin Burns, Ryan Chadha, Pablo Moreno Franco

**Affiliations:** 1 Department of Critical Care, Mayo Clinic, 4500 San Pablo Road, Jacksonville, FL USA; 2 Department of Anesthesiology, Mayo Clinic, Jacksonville, FL USA; 3 Department of Surgery, Mayo Clinic, Jacksonville, FL USA

**Keywords:** Case report, Liver transplantation, Cirrhosis, Air embolism, Intracardiac thrombus

## Abstract

**Background:**

Intracardiac thrombus and vascular air embolism represent rare complications in the context of orthotopic liver transplantation. While isolated reports exist for intracardiac thrombus and vascular air embolism during orthotopic liver transplantation, this report presents the first documentation of their simultaneous occurrence in this surgical setting.

**Case presentation:**

This case report outlines the clinical course of a 60-year-old white female patient with end-stage liver disease complicated by portal hypertension, ascites, and hepatocellular carcinoma. The patient underwent orthotopic liver transplantation and encountered concurrent intraoperative complications involving intracardiac thrombus and vascular air embolism. Transesophageal echocardiography revealed the presence of air in the left ventricle and a thrombus in the right atrium and ventricle. Successful management ensued, incorporating hemodynamic support, anticoagulation, and thrombolytic therapy, culminating in the patient’s discharge after a week.

**Conclusions:**

This report highlights the potential for simultaneous intraoperative complications during orthotopic liver transplantation, manifesting at any phase of the surgery. It underscores the critical importance of vigilant monitoring throughout orthotopic liver transplantation to promptly identify and effectively address these rare yet potentially catastrophic complications.

## Background

End-stage liver disease (ESLD) disrupts the coagulation system by reducing procoagulation and anticoagulation factors, causing qualitative and quantitative platelet defects and reducing fibrinolytic proteins levels [1]. This disturbance results in a fragile coagulation/anticoagulation equilibrium prone to tipping toward either bleeding or clotting [1]. Despite this vulnerability, the estimated incidence of thromboembolic complications, such as intracardiac thrombosis (ICT), during orthotopic liver transplantation (OLT) remains relatively low [2–4]. The pathogenesis of ICT formation during OLT remains elusive, yet its development can have catastrophic consequences and has been associated with poor survival outcomes [5].

Complications such as vascular air embolism (VAE) can also manifest during OLT. This complication arises from the entrainment of air from the operative field into the venous circulation or intrahepatic air in the donor’s liver. VAE poses a threat to hepatic microcirculation and cardiac output (CO) and has the potential to traverse from the right side of the heart to the pulmonary and systemic circulation. If air emboli reach the left side of the heart or the systemic circulation, it is termed paradoxical air embolism (PAE), and could lead to air embolization in the terminal arterial bed of the brain and heart, resulting in cardiac and neurological complications. This case report details the simultaneous occurrence of intraoperative ICT and VAE during the anhepatic phase of OLT, identified through transesophageal echocardiogram (TEE) in a patient with cirrhosis.

## Case presentation

The patient, a 60-year-old white female with ESLD secondary to alcoholic cirrhosis, underwent OLT. Her medical history included hypertension and breast cancer treated with chemoradiation and bilateral mastectomy 13 years prior. ESLD complications encompassed portal hypertension, ascites, hyponatremia, and hepatocellular carcinoma managed with transarterial radioembolization with yttrium-90 (Y-90 TARE). Preoperative assessments, including transthoracic echocardiogram (TTE), myocardial perfusion imaging, and electrocardiogram were unremarkable, except for a grade 3/3 diastolic dysfunction on the TTE and an intrapulmonary shunt. The patient had a biologic model for ESLD (MELD-Na) of 21 (no dialysis, creatinine 0.76 mg/dL, bilirubin 6.5 mg/dL, international normalised ratio (INR) 1.4, sodium 134 mEq/L, and Child–Pugh class B (bilirubin > 3 mg/dL, albumin 2.8–3.5 g/dL, INR < 1.7, slight ascites, and no encephalopathy) at transplantation.

In the operating room, the patient was positioned supine. Anesthesia induction involved fentanyl and propofol and was maintained with sevoflurane without complications. Muscle relaxation was achieved with rocuronium, and ventilation was mechanically controlled to maintain an end-tidal carbon dioxide pressure (ETCO_2_) between 30 and 35 mmHg. Intraoperative monitoring included standard American Society of Anesthesiologists monitors and a 20-gauge right and left radial artery invasive monitor. Continuous pulmonary artery and central venous pressure measurements were obtained via a Swan–Ganz catheter placed through a 9 French multi-lumen sheath introducer in the right internal jugular vein.

There was a significant increase in time, technical difficulty, and physical effort required to complete the transplant due to hypertrophy of the caudate lobe, encircling and wrapping the inferior vena cava (IVC). During dissection, an IVC injury occurred, requiring emergent clamping.

**Fig. 1:**
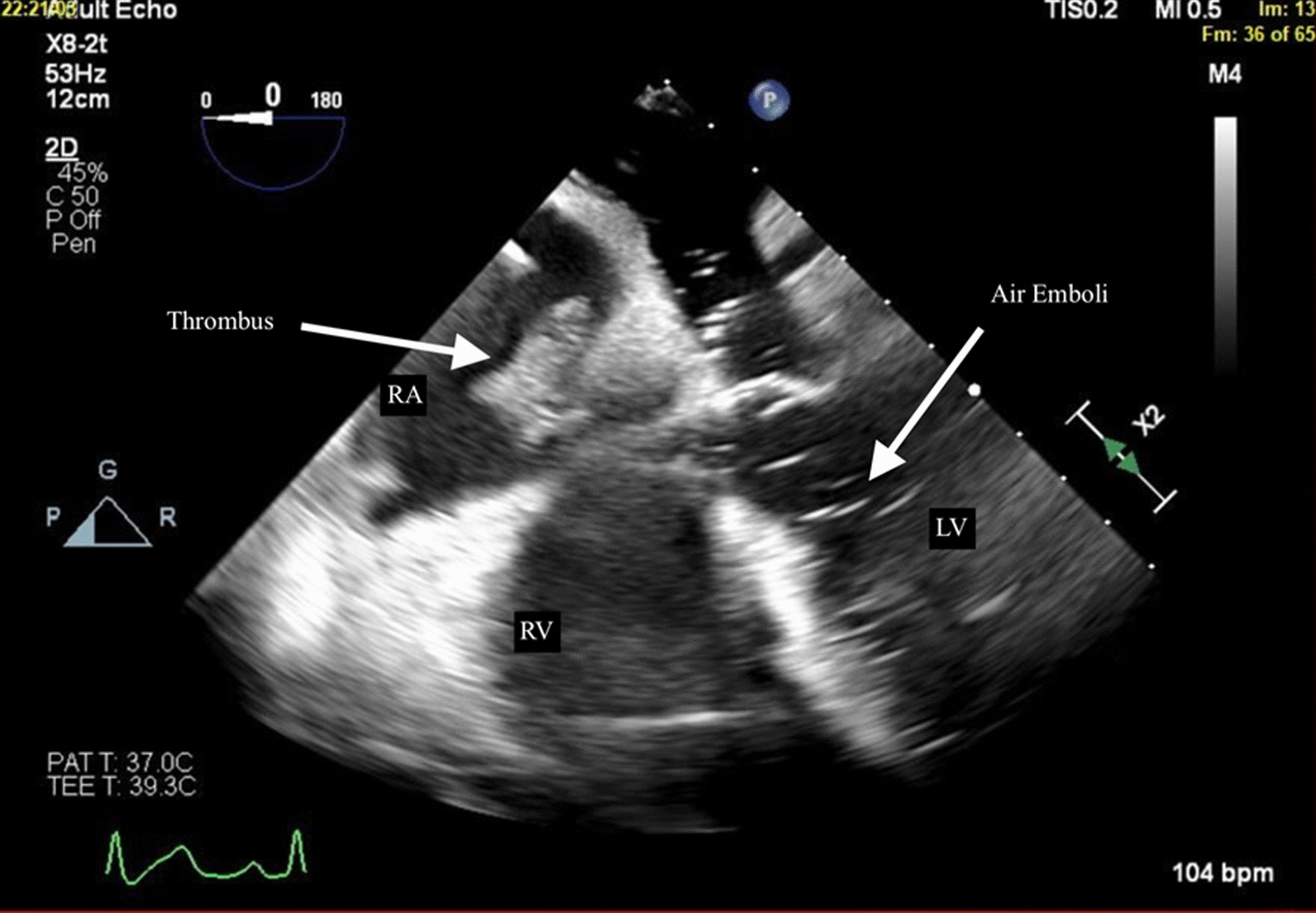
Four-chamber view demonstrating thrombus (left arrow) in the right atrium (RA) and air emboli (right arrow) in the left ventricle (LV). *RV* right ventricle

Upon clamping, there was a sudden increase in mean pulmonary arterial pressure (mPAP) with decrease in the mean arterial pressure, increased central venous pressure, reduced ETCO_2_ (from 35 to 20 mmHg), tachycardia, and decreased oxygen saturation. The patient received epinephrine boluses with real-time titration of an epinephrine drip. Transesophageal echocardiography (TEE) revealed air in the left ventricle (LV), consistent with an air embolism (Fig. 1). Upon IVC clamp release, TEE revealed a thrombus in the right atrium and right ventricle (RV) (Fig. 1). Despite an initial intravenous bolus of heparin (5000 units), the thrombus continued to increase in size, prompting administration of low doses of intravenous recombinant tissue plasminogen activator (rtPA) with further resolution of the thrombus and normalization of the hemodynamic parameters. The first dose of rtPA comprised a 1.5-mg intravenous bolus, followed by an additional 0.5 mg. Although air persisted for 5 minutes after IVC repair and unclamping, the patient remained hemodynamically stable and without signs of systemic embolism. Both the ICT and VAE resolved, demonstrating normal RV/LV function at the conclusion of the case. During the surgery, the patient received 12 units of packed red blood cells, 14 units of fresh frozen plasma, four pools of platelets, and four cryoprecipitate pools. The blood product resuscitation was guided by thromboelastometry, hemoglobin level, and estimated blood loss. Postoperatively, the patient recovered well without complications and was discharged home after a week.

## Discussion

OLT stands as the gold standard treatment for patients with ESLD. Despite continuous enhancements in surgical techniques and medical technology that have contributed to increased safety, the procedure is not devoid of risks, and intraoperative complications persist. A notable risk for patients undergoing OLT is the heightened susceptibility to intraoperative bleeding owing to compromised hemostatic balance. Rare complications, such as ICT and VAE, have been documented [2–4]. However, the simultaneous occurrence of ICT and VAE during OLT remains uncharted in existing literature.

VAE, a rare complication during or after liver transplantation, is predominantly reported during the pre-anhepatic and reperfusion phases. In this case, our suspicion points to the iatrogenic defect in the IVC during dissection as the likely entrainment source. However, it is prudent to consider an alternative hypothesis regarding the Swan–Ganz catheter placement, acknowledging the potential for air entry during this intraoperative procedure. The impact of VAE is contingent on both the volume and rate of air entry. The patient’s tachycardic response likely resulted from both right heart strain induced by the air embolism and as a reflex response to hypotension. The hypotension, in turn, could be attributed to reduced CO, while the increase in mPAP resulted from the decline in CO and increased filling pressures. Furthermore, the decline in CO can provoke a ventilation/perfusion mismatch, causing reductions in ETCO_2_ and oxygen saturation.

Patients with ESLD, with their high prevalence of pulmonary abnormalities, face elevated risks associated with VAE. In this demographic, intrapulmonary shunting may arise from either intrapulmonary vascular dilation at the precapillary level or direct arteriovenous communications, making paradoxical air embolism (PAE) possible even in the absence of intracardiac abnormalities [6]. While the exact point of air bypassing the pulmonary vasculature could not be pinpointed in our case, the preoperative TEE findings suggest that the air likely traversed intrapulmonary shunts. PAE has the potential to induce severe cardiac and neurological complications; however, our patient did not suffer any adverse effects.

TEE stands out as the most sensitive monitoring tool for detecting VAE during OLT; however, a standardized approach for its diagnosis is lacking. Crouch *et al.* demonstrated a lack of uniformity in the practice of monitoring during OLT, with 49% of US liver transplantation centers employing routine use of TTE and 18% reserving its use only for specific circumstances [7]. Effective management of VAE and its PAE hinges on prompt identification and correction of the air embolus source, along with hemodynamic support and prevention of further air entrainment. In our case, the patient was managed with immediate repair of the IVC defect, and hemodynamic support was provided through the administration of vasopressors.

ICT is another recognized complication occurring throughout various phases of OLT, with a predilection for the pre-anhepatic and post-reperfusion phases [3, 8]. While ICT incidence is rare (0.36–6.2%), its intraoperative mortality rate can reach 68%, underscoring the severity of this complication despite interventions [2–4]. The utilization of TEE during OLT may influence ICT outcomes, as indicated by Fagelman *et al.*, who reported higher incidence and lower mortality with routine TEE use, suggesting its potential role in early recognition and treatment, thereby mitigating the risk of intraoperative cardiac arrest and reducing mortality [8].

Numerous risk factors have been associated with ICT development, with reported culprits including veno–venous bypass and antifibrinolytic medications; however, our case did not involve the use of either [4]. Additional risk factors in our patient included alcohol-related cirrhosis, a history of malignancy, and the transfusion of platelets, fresh frozen plasma, and cryoprecipitate during surgery [2]. Additionally, caval clamping and myocardial stunning from the air embolism may induce a low-flow state, contributing to right heart and central venostasis, further predisposing to thrombus formation. The clinical presentation of ICT during OLT is influenced by TEE utilization. In centers without routine TEE use, intraoperative cardiac arrest is the primary presentation, whereas sudden hemodynamic instability and incidental finding of ICT are more common with routine TEE use [8].

Currently, TEE remains the sole confirmatory tool for diagnosing ICT; however, its standardized use during OLT varies. Treatment strategies for ICT also lack consensus, with different approaches, such as intravenous heparin [3, 9], rtPA [3, 10], and thrombectomy [4] reported. The decision to use intravenous heparin, thrombolytic therapy, or a combination depends on factors like thrombus size, location, hemodynamic status, and the risk of coagulopathic hemorrhage post-treatment. In our case, initial treatment with a heparin bolus proved insufficient, leading us to administer low dose of rtPA, resulting in rapid hemodynamic improvement and thrombus dissolution without complications, aligning with our prior successful experiences.

## Conclusions

This case highlights the effective management of concurrent intraoperative complications involving ICT and VAE during OLT. It emphasizes the critical significance of vigilant monitoring and timely interventions in liver transplantation surgeries to effectively address and avert potential complications, thereby ensuring optimal outcomes for the patient.

## Data Availability

Not applicable.

## Abbreviations

CO: Cardiac output
ESLD: End-stage liver disease
ETCO_2_: End-tidal carbon dioxide
ICT: Intracardiac thrombus
IVC: Inferior vena cava
LV: Left ventricle
mPAP: Mean pulmonary artery pressure
OLT: Orthotopic liver transplantation
PAE: Paradoxical air embolism
RV: Right ventricle
rtPA: Recombinant tissue plasminogen activator
TEE: Transesophageal echocardiogram
TTE: Transthoracic echocardiogram
VAE: Vascular air embolism
